# Insights into the Slow Uptake of Residential Lead Paint Remediation Funds: A Lancaster, Pennsylvania, Case Study

**DOI:** 10.3390/ijerph18020652

**Published:** 2021-01-14

**Authors:** Margaret Cherney, Sarabeth Erdman, Madeline Kuon, Nicholas Shupin, Najeda Regis, Emma Fitzelle-Jones, Kylie Givler, Susan Baldrige, Harriet Okatch

**Affiliations:** 1 NORC at the University of Chicago, 4350 East West Highway, 8th Floor, Bethesda, MD 20814, USA; mcherney@fandm.edu; 2 District of Columbia Sustainable Energy Utility (DCSEU), 80 M Street SE, Suite 301, Washington, DC 20003, USA; serdman@fandm.edu; 3 Biology Department & Public Health Program, Franklin and Marshall College, 415 Harrisburg Avenue, Lancaster, PA 17603, USA; mkuon@fandm.edu (M.K.); nshupin@fandm.edu (N.S.); nregis@fandm.edu (N.R.); 4 School of Public Health, Boston University, 715 Albany St, Boston, MA 02118, USA; efitzell@bu.edu; 5 Department of Physical Therapy, University of Delaware, 210 South College Ave, Newark, DE 19716, USA; kgivler@udel.edu; 6 Partnership for Public Health, 333 N Arch St, Lancaster, PA 17603, USA; Susan.Baldrige@lanchc.org

**Keywords:** childhood lead poisoning, knowledge levels, blind trust, HUD grant, Lancaster, PA, residential lead paint remediation, social media

## Abstract

Lead poisoning is a preventable condition that continues to affect thousands of children each year. Given that local governments and municipalities are eligible to apply for federal funds to perform lead remediation in low-income family homes, we sought to understand how lead poisoning knowledge levels may affect the uptake of these funds. We recruited and conducted 28 in-depth, semi-structured interviews with community members from Lancaster County in the state of Pennsylvania in the USA. We audio-recorded and transcribed each interview, and analyzed each transcript for salient themes. The interviewed participants displayed a varying degree of knowledge about lead and lead poisoning. Most of the participants were unaware of the lead paint remediation funds. Participants learned about lead from various sources, such as social media, and personal experiences with lead poisoning appeared to enhance knowledge. Some participants assumed lead poisoning prevention would be addressed by other stakeholders if necessary, including healthcare professionals and landlords. The results of this study suggest that in order to increase the timely uptake of the remediation funds, community-based organizations should design interventions that aim to increase awareness and knowledge about lead poisoning and lead poisoning prevention. These interventions should be tailored for different audiences including community members, healthcare professionals, and landlords.

## 1. Background

Childhood lead poisoning is a pervasive issue in the United States, affecting the lives of approximately half a million children ages six and under [1]. In 2014, the water contamination crisis in Flint, Michigan, brought the issue of lead poisoning to the attention of the mainstream media and into the minds of the public [2]. The Centers for Disease Control and Prevention has not determined a safe blood lead level and recommends that children with blood lead levels greater than 5 µg/dL receive case management services [3]. Childhood lead exposure has been linked to brain and nervous system damage, delayed growth and development, and learning and behavioral challenges [4]. Elevated blood lead levels have been associated with lower educational attainment, antisocial behavior, and higher hyperactivity scores in children [5]. Disparities in childhood lead poisoning illustrate the inequities that persist in society, as lead poisoning disproportionately affects low-income, minority, and refugee communities [6,7].

While lead poisoning is both a national and global concern, Lancaster County, in the state of Pennsylvania, USA, provides a notable case study for this issue. In 2018, Lancaster County had a significantly higher rate of childhood lead poisoning compared to the state of Pennsylvania (6.80% vs. 4.09%) [8]. Lead-based paint was banned in the United States in 1978; in Lancaster County, 27% and 59% of homes were built before 1950 and 1980, respectively [9]. For this reason, attempts to mitigate childhood blood lead levels in Lancaster County are often focused toward lead paint remediation in older homes. In addition to lead paint, low testing rates in Lancaster contribute to untreated lead poisoning. In Lancaster, only 9.89% of children under the age of six years received a blood lead test in 2017 [10]. Limited funding in relation to the number of homes with lead-based paint is often a key challenge in performing lead remediation work [11]. In 2016, the United States Department of Housing and Urban Development (HUD) awarded Lancaster County a $1.33 million Lead Hazard Control (LHC) grant [12]. This grant provided funding to support lead paint remediation efforts in low-income homes in which a child under the age of six resided, or visited for more than six hours a week.

Despite the acquisition of HUD LHC funds, rates of lead poisoning remain high and uptake of HUD grant funds remains slow. The health belief model [13] outlines that for individuals to act on a health concern, they must have a perceived susceptibility and severity, as well as a sense of perceived benefits. In the presence of these factors, a cue to action and self-efficacy are required for beneficial behavioral change. This study aimed to investigate caregivers’ knowledge levels of lead poisoning, the factors that influence those levels, and reasons for the initially slow uptake of the HUD funding. Our main goal was to analyze parents’ sources of information about lead, concerns about lead exposure, and personal experiences with lead poisoning. In doing so, we ascertained how and when individuals obtained information and whether their knowledge increased their willingness to abate their homes of lead paint. This information will provide insight into how health messaging and advertisements for future lead remediation funding can truly reduce the burden of lead poisoning.

## 2. Materials and Methods

Through this study, approved by the Institutional Review Board of Franklin and Marshall College, we aimed to understand the factors that contributed to the initial slow uptake of the HUD LHC grant awarded to Lancaster County, Pennsylvania, for residential lead remediation.

In June 2016, the City of Lancaster in the state of Pennsylvania, USA, was one of 23 recipients (1 health department, 11 cities, 9 counties, and 2 states) representing 15 states that received funding from HUD to remediate homes: a total of $52.6 million was disbursed. The City of Lancaster received $1.33 million dollars to remediate lead-based paint in 100 homes over a period of three years. About $700,000 dollars were to be administered by the City of Lancaster in Lancaster County and $600,000 was to be administered by Lancaster County Housing and Redevelopment Authority to remediate lead-based paint in homes in the city and outside the city (but within the county), respectively. The funds were available to anyone (tenant, homeowners living in the homes, and landlords) who fit the low-income definition as defined by HUD; for landlords, the income eligibility was determined by the income of the tenants. Landlords were required to contribute towards the remediation costs. In June 2017, a local Lancaster newspaper reported that only two households had applied for the funds [14].

Between February and May 2018, researchers trained in qualitative interviewing techniques recruited participants from two Head Start locations, a Healthy Beginnings Plus office for pregnant women receiving medical assistance, the Women, Infants and Children (WIC) Nutrition Program office, and through door-to-door solicitation in downtown Lancaster. The research team selected these locations due to the likelihood that individuals frequenting these venues would meet the eligibility criteria for both this study and the HUD grant. Individuals over the age of 18 years who were caregivers of a child or children under the age of six years, including pre-natal, were eligible to participate in this study. Additionally, through word of mouth referrals from key informants, we contacted several landlords. However, only two landlords were interested in participating in the study.

Researchers reviewed the study aims and consent process with all interested individuals prior to conducting semi-structured interviews with all consenting participants. Interviews included between one to three participants. At least two researchers were present for each interview. The primary interviewer asked the protocol questions and any appropriate probing questions, while the secondary interviewer took notes and asked clarifying questions. Participants also completed a brief demographic data collection survey. Contingent upon participant consent, interviewers audio-recorded the conversations to ensure transcript accuracy. Interviews lasted between 5 and 35 min. Research staff conducted the majority of interviews in English. One interview was conducted in Swahili with the help of a certified interpreter.

Each interview was concluded with a debrief session: the primary interviewer provided the participant(s) with an educational infographic document outlining sources of lead, strategies to mitigate lead consumption and exposure, and how to interpret blood lead test results. Information about lead poisoning prevention resources available to Lancaster residents was shared with the participants. Interviewers reviewed this document with each participant and answered any follow-up questions. Participants were given a $20 Visa gift card at the end of the interview for their participation.

Research team members transcribed most audio files (*n* = 23) and QSR International’s NVivo Transcription [15] services transcribed the remaining files (*n* = 5). Research staff reviewed transcripts (*n* = 28) against their corresponding audio file to ensure accuracy. Researchers implemented deductive methods to develop an initial set of codes, tested these codes with a sample of the study data, and revised the set of codes using inductive methods. The principal co-investigators (M.C., S.E.) participated in three rounds of coding to achieve a final Kappa score of 0.82 (Round 1 Kappa score = 0.24; Round 2 Kappa score = 0.75). After achieving a sufficient Kappa score, the principal co-investigators coded the transcripts using QSR International’s NVivo 12 software [15] and analyzed the data by theme.

Since only two landlords were interviewed and saturation was not reached, these transcripts were excluded from the analysis.

### Ethical Considerations

The study and the corresponding materials, including the consent form and interview protocols, were approved by the Institutional Review Board of Franklin and Marshall College (Code: #R_xo4vZpN8x1u0hJD). All participants gave consent to be part of the study by signing a consent form detailing the purpose of the study and the potential benefits and risks. All participants consented to having their interview audio-recorded and also consented to the use of their data in an aggregated and de-identified format.

## 3. Results

The demographic characteristics of the 33 participants from the 28 interviews are summarized in Table 1. Not all participants provided information for each demographic question. The participants ranged in age from 19 to 56 years old: mean age 31.94 years (standard deviation (SD) 9.99). The sample consisted of 90% females (*n* = 28). Over one-third of participants (38.7%, *n* = 12) identified as white and almost one-third of participants (32.3%, *n* = 10) identified as black. The average number of children per participant was 2.29 (SD 1.18), multiple participants were pregnant and one participant was caring for their grandchild. More than one quarter (26.6%, *n* = 8) of the sample had attained post-secondary education. The majority of participants (88.5%, *n* = 23) reported earning an annual salary of less than $40,000. Additionally, the majority of the participants (79.3%, *n* = 23) were renting the property in which they lived. Most participants (60%, *n* = 18) did not know the age of the house or if the house had lead-based paint; however, over half of the participants (57.1%, *n* = 16) observed chipping, peeling, and cracking paint in their homes. The coding and analysis of the interview transcripts revealed several themes described below, all related to knowledge about lead poisoning.

### 3.1. Knowledge

Each participant discussed their level of knowledge regarding lead and its health effects. Participants provided both accurate and inaccurate responses to questions concerning sources of lead. Water, paint, and toys were the most commonly reported accurate sources of lead, while pens and pencils were the most commonly reported inaccurate sources of lead. Some participants were unaware of lead, its health effects, and/or the HUD grant, while others initially reported low levels of lead knowledge yet provided accurate information about lead later in the interview. For example, when asked what they know about lead, one participant stated “*nothing, really*”, but when asked about potential sources of lead the participant cited paint as a potential source. Conversely, some participants reported familiarity with lead and were highly confident in their knowledge of the subject, yet they reported inaccurate information. For example, some participants were confident that pencils were one of the main sources of lead, even though that information is inaccurate.

Knowledge was a ubiquitous theme, including a lack of knowledge, inaccurate knowledge, and accounts of how knowledge influenced participants’ actions and concerns. The data revealed complex insights concerning the influence of knowledge, or lack thereof, on participants’ intentions to address lead poisoning and apply for the HUD funding. There are factors that influence knowledge, including sources of information, concerns, and experiences, and factors that are influenced by knowledge, including actions and expectations. Participants directly and indirectly discussed the multifaceted influence of knowledge on their awareness, and concern, of lead poisoning throughout their responses.

### 3.2. Sources of Information

Participants identified a variety of sources from which they gained knowledge on lead and its health effects. Participants commonly mentioned the following sources: social media, medical providers’ offices, offices of service providers such as WIC, friends, and family. For example, when asked from where they learned about lead, a participant stated:

“…through WIC and the family doctor is where, but I didn’t know as much until I became a mom myself about the testing and when to do it. But actually, on Facebook there is a girl who also rents, and her child had very high levels and it was very dangerous and she had to go to the hospital and they’d keep an eye on that child because the levels [were] way out of control, it was the house they were renting.”

This response highlights social media as a source of information and demonstrates the impact of reading or hearing stories of individuals who have experienced lead poisoning. When asked the same question, another participant stated:

“Um, I guess just from, uh, from having kids just kind of hearing about it from schools and uh, just to be aware of it from doctors and pediatricians and stuff like that.”

These responses capture a variety of both the formal and informal sources of information participants commonly referenced.

### 3.3. Lead Exposure Concerns

Interviewers asked participants if they were concerned that their child(ren) may be exposed to lead. Participants’ lead concerns were frequently associated with water quality. Multiple participants expressed concern about their water quality, particularly when asked about their knowledge of the lead-contaminated water in Flint, Michigan. An interviewer asked a participant aware of the lead poisoning that took place in Flint if they were concerned about their local water, to which the participant stated:

“Yeah we don’t drink it. I buy, well that doesn’t mean anything, but I buy bottled water and I, even my daughter, I don’t give her faucet [water], I buy her baby water. We don’t drink faucet water. But what’s the difference because we’re bathing in it, and what’s the difference because we cook with it, which I never really thought about. I’ll boil potatoes in it, but I won’t drink it. That’s weird.”

When asked if their decision to prioritize consuming bottled water was due to lead concerns or other health issues, the participant replied:

“Um, just lead and you just don’t know what’s in the water nowadays but probably lead [is] not the biggest concern, it’s just I don’t drink water, it tastes funny anyway and with them talking about bringing a pipeline to Lancaster, I don’t know, I buy bottled water.”

Participants frequently mentioned water quality concerns as opposed to other sources of potential lead exposure, such as soil.

In some instances, participants expressed concern and suggested that their concerns about lead were initiated by their participation in the study interview. For example, when asked if they were concerned that their child may be exposed to lead, one participant stated:

“Right now, yes, because you’re doing a study it makes me wonder what’s going on. I am going to research it after I get out of here, ask a bunch of questions about it.”

### 3.4. Lead Poisoning Experiences

While participants shared their general knowledge and concerns regarding lead and lead poisoning, they often mentioned personal experiences that influenced their lead poisoning knowledge levels. Several participants shared stories describing the effects of lead poisoning they had seen in their own children or family members’ children. Participants described observing symptoms of lead poisoning, such as speech delays, hyperactivity, excessive crying, and attention deficit hyperactivity disorder (ADHD). Furthermore, participants described lead poisoning as something that they were unaware of until it had affected their family. One participant reflected on the experience of her son having high lead levels, and said:

“But now that my son has it, I’m like searching stuff up online, like I’m real big on it now.”

She continued to share how her experience increased her knowledge of lead poisoning and the threat it poses, which she has since used to bring awareness to friends, stating:

“As a matter of fact, I was just talking to my friend about it, because she was like ‘My daughter’s real bad’ and I was like ‘Oh because my son has behavioral issues too, why don’t you get her tested for lead.’ Cause she just moved into her own place and stuff, so I was like ‘Yeah get your place checked, go to your doctor and get her tested and stuff, see if she got it and stuff.’”

### 3.5. Blind Trust

Following lines of questioning regarding lead exposures, concerns, and testing, a theme of blind trust emerged among some participants. These participants stated that they had assumed if lead poisoning was an issue that warranted concern, then someone would have made them aware. Participants shared their trust in doctors to test children for anything important without prompting, such as blood lead levels. Many also noted their belief that lead exposure was no longer an issue since lead was removed from paint several decades ago.

When asked about her child’s exposure to lead and the child’s testing history, one participant stated:

“Don’t the doctors like check for lead and stuff? That’s what I would’ve thought that the doctors check for it because I wouldn’t know for a fact like I wouldn’t know unless the doctors would tell me?”

Several other participants described a trust in their children’s doctors to test for lead levels, without prompting, if it was in fact an issue that warranted concern. Participants also trusted landlords to take initiative and precautions to avoid dangers associated with potential sources of lead in the home.

A female caregiver explained this trust, saying:

“Ok, yeah, and we have a pretty good landlord so I think he would be up on it, he’s not like a slumlord who’s just like ‘uh we don’t care- get you outta there and get somebody else in there, he’s not like that.”

Another participant described her lack of awareness of lead levels in Lancaster and assumption that lead exposure was no longer an issue to keep in mind, explaining:

“I’m not aware of any, anything like that. I think it’s because they say it’s supposed to be banned I guess and you’re supposed to change it but I don’t worry about it because I expect people that rent or, you know, have their own homes to actually look for, go to Lowe’s and look for lead-free paint.”

These responses help to explain the low levels of testing in Lancaster, as well as the low levels of interaction with the HUD grant.

### 3.6. Knowledge as a Cue for Action

The information shared during the debrief session was well-received by participants—participants unanimously noted that the information provided was extremely helpful. Additionally, participants shared that this newfound information would lead them to actions, such as thinking of their child’s potential exposure to lead on a daily basis, actively sharing information with friends and family, remediating homes of lead, and getting their child(ren) tested. Most participants expressed guilt and surprise that they had not known about lead poisoning earlier and were eager to share their learned knowledge with friends and family.

One participant, who was in the process of applying for the HUD grant, indicated that the information would motivate her to urgently complete the grant application. This participant said:

“Yeah, yeah I will actually complete the form and send it in to do what I have to do in my home. Definitely.”

Uptake of the HUD grant may be facilitated by increasing awareness of the effects of lead poisoning and associated mitigation practices.

### 3.7. Residential Lead Paint Remediation Grant

A few participants knew about the HUD LHC grant. These participants either had a child who had been diagnosed with a high blood lead level, and hence had undergone a home inspection and abatement as per the current guidelines, or they knew of someone who had their home remediated through the HUD LHC program. One participant, whose child had been diagnosed with an elevated blood lead level, and whose home had been recently abated, did not know that these funds were available, nor did they know the eligibility criteria. Some participants, who had never heard of the HUD LHC grant, were interested in applying for the funds. However, a few were not interested because they trusted the landlord would address the problem, or they visually assessed the paint in the home and concluded it was in good condition because there was no chipping, peeling, or cracking paint.

The majority of participants, even those with high lead poisoning knowledge levels, had never heard of the HUD LHC grant for lead abatement. Upon learning about the grant, most of these participants, predominantly renters, assumed that they would not meet the eligibility criteria. They thought only landlords or homeowners could apply and expected they would need the permission of the landlord to apply for these funds. This assumed criteria affected the interest of many participants, as they believed their landlords would not be interested in participating in the process. Additionally, some participants were concerned about how much they would have to contribute to the cost of the lead abatement.

Generally, participants’ responses supported the following reasons for why the general population was not applying for the funds: residents do not know about the grant and the eligibility criteria, and people do not know if they have lead in their home, as illustrated by this quote from a participant “*they [Lancaster residents] probably don’t know that it [the HUD LHC grant] is available, or they don’t know about the lead in their homes.*” Furthermore, one participant highlighted that the uncertainty and unanswered questions about the abatement process could contribute to initial slow uptake rates, stating:

“If it’s free, that would be basically it, like, if the pricing, like is it free. Um, the convenience of it, like do we have to leave the home? Is it a one-day thing or is it over several days? How long is it going to take? Are we allowed to come back in the home? You know, stuff like that.”

## 4. Discussion

The findings of this study suggest that lead knowledge levels vary among Lancaster residents: some participants had impressive lead knowledge levels, and others did not know much about lead. The low lead knowledge levels observed in our study are consistent with previous qualitative and quantitative studies [16,17,18,19]. Interestingly, in our study, a subset of individuals who considered themselves knowledgeable about lead reported inaccurate information about the subject. This subset of individuals may be at a heightened risk of lead poisoning because they may be less likely to advance their knowledge or pay attention to messaging and outreach about lead and the HUD LHC grant to remediate their homes. This presents a unique challenge for public health practitioners. Public health efforts should aim to identify incorrect knowledge and develop educational interventions to disseminate accurate knowledge on lead and lead poisoning. Dissemination of information will also develop residents’ confidence and self-efficacy to adopt preventive measures and to share information.

Such information dissemination should employ conventional media, such as television, radio, and newspapers, that have been observed to be effective for motivating changes in health behaviors such as tobacco use, condom use, physical activity, and sun protection [20,21,22,23]. It is worthy to note that none of the participants in our study mentioned these avenues as sources of lead poisoning information. Rather, participants mentioned more personalized sources of information including physicians, social service organization representatives, social media, and friends and family who have experienced lead poisoning. Since the accuracy of information from some of these sources is unregulated and cannot be verified, public health messaging should adopt various readily accessible media for lead poisoning and prevention information dissemination. For example, in Hartford, Connecticut, a public–private partnership utilized billboards, signs on city buses, bus shelters, sanitation trucks, and milk cartons, and created videos and an art display to promote lead poisoning prevention. These efforts resulted in 45% of the population taking action to prevent lead poisoning [24].

The reliance on social media as a source of information might explain why participants were more concerned about lead in water and not in paint. Water quality was a significant concern. We attribute this concern level to the publicity of the Flint water crisis; nationwide, in the first six months of 2016, there were about 750 articles in the top five newspapers and over 2.1 million tweets about this crisis [25]. This increased publicity may have influenced participants’ concerns regarding lead in water. However, a report by the Environmental Protection Agency and other researchers notes that most lead poisoning cases are attributed to lead-based paint, rather than water [26,27,28,29]. Because sources of exposure to lead can differ over time and across geographical areas, comprehensive and continual lead exposure education is essential to reducing the incidence of lead poisoning. Before the water crisis in Flint (January–September 2013), 2.4% of children had elevated blood levels. During the same time frame in 2015, after the switch in water source, this rate doubled to 4.9% [30]. In comparison, in the calendar years 2013 and 2015, the rates of lead poisoning among children tested in Lancaster County were 8.7% and 8.9%, respectively [31,32].

As is the case with other diseases, such as sexually transmitted infections and breast cancer [33,34,35], we found that caregivers who had personal experiences with lead poisoning were more likely to want to learn more about lead poisoning and the available funds for lead remediation. Usually, personal experiences with lead poisoning indicate a missed opportunity for primary prevention and hence the need for the use of different media to increase awareness is apparent. Among those with no exposure, it is likely that optimistic bias is at play. Individuals believe that negative things are more likely to happen to others, which subsequently leads to the underestimation of one’s own risk [36]. This was observed in a study by Wolde et al., in which 80.35% of Jersey City residents felt they were at a lower risk of exposure to lead-based paint compared to the average New Jersey state resident, even though the rate of lead poisoning in the state of New Jersey is lower than the rate of lead poisoning in Jersey City [37]. Public health practitioners are challenged with the design of interventions to address this unrealistic optimism and to increase primary prevention of lead poisoning through the uptake of funds for residential lead paint remediation.

Participants in the study were confident that other entities (physicians, landlords, and the government) would be invested in their health and well-being. Blind trust has been observed with other health conditions, such as seasonal influenza, where patients are more likely to get the seasonal influenza vaccine if their physician recommends it [38]. In a study assessing patient–physician relationships, 30% of individuals reported completely trusting their physicians to put their medical needs above all considerations, while only 10% did not trust their physician at all [39]. Yet, studies show that physicians do not necessarily follow blood lead level screening guidelines. In a Wisconsin study of Medicaid-enrolled children, only 32% of children received the recommended blood lead tests [40]. Medicaid guidelines mandate universal screening of all enrollees at 12 and 24 months. In another study, 48% of doctors in Nevada did not strictly adhere to testing guidelines [41]. These studies, coupled with our findings, suggest that individuals who completely trust and rely on their physician’s prompting might not be receiving adequate blood lead testing. Therefore, they are not accessing the information about lead poisoning prevention that would accompany the test. To improve communication about lead poisoning prevention and treatment, public health efforts should be collaborative and target physicians, educators, and landlords. In their studies, Gettens et al. and Phoenix et al. demonstrate the efficacy of educational interventions addressed at the medical community and real estate community, respectively [42,43]. Such efforts will likely increase rates of primary and secondary prevention of lead poisoning.

Our findings also demonstrate that after the debriefing, when interviewers shared detailed information about lead poisoning exposure, effects, testing, prevention, and the HUD LHC grant, some individuals were immediately motivated to act. This is aligned with the findings from Kegler and Malcoe’s study, which demonstrated that individuals with higher lead knowledge levels also possessed higher self-efficacy to adopt preventive measures including hand washing, having children play on safe surfaces, and cleaning with a damp cloth [44]. Similarly, in a study measuring breast cancer screening rates, individuals with a high knowledge level of breast cancer were more likely to get screened [45]. This suggests that interventions targeting increased lead knowledge levels, coupled with information about the availability of the remediation funds, can potentially increase the motivation to remediate homes with lead-based paint. Interventions to increase the use of the HUD LHC grant funds can adopt a social marketing approach to disseminate this information [46,47].

This is the first study of this nature that has been conducted in the City of Lancaster, a city that has had a consistently high prevalence of lead poisoning among the small proportions of individuals who receive testing. The qualitative nature of the study limits the generalizability of the findings; however, public health practitioners in Lancaster can utilize these findings to develop contextually appropriate interventions to increase awareness about the HUD LHC grant and residents’ lead poisoning knowledge. Future communications should address lead exposure, effects, testing, and prevention, and should target a variety of groups including physicians, social service professionals, landlords, educators, and residents.

## 5. Conclusions

The findings of the interviews with Lancaster residents suggest that knowledge levels about lead poisoning sources, effects, and preventive practices vary. The key factors that emerged as influencing lead knowledge were experiences with lead and concerns about lead. Information about lead and lead poisoning was obtained from multiple sources including family members, healthcare providers, social welfare service providers, and social media. On the other hand, these interviews revealed that information about the HUD LHC grant is not reaching most of the population who would benefit from residential lead remediation: providing knowledge about lead poisoning and/or the HUD LHC grant served as a cue for action. The individuals who were not motivated by the HUD LHC grant are likely trusting other responsible groups, such as physicians and landlords, to address the issue. The findings suggest a need for tailored interventions and indicate potential use of a social marketing framework may be helpful to increase knowledge about lead. The development and design of interventions, in collaboration with local governments and community-based organizations, may increase uptake of funds and primary prevention of childhood lead poisoning. Furthermore, these interventions should be tailored to different audiences including healthcare professionals, landlords, and early childhood educators.

## Figures and Tables

**Table 1:** Demographic characteristics of participants.

Characteristic	*n*	Percent	Mean; SD
Age (*n* = 31)			31.94; 9.99
Gender (*n* = 31)			
Male	3	9.7%	
Female	28	90.3%	
Marital Status (*n* = 30)			
Single	21	70%	
Married	6	20%	
Co-habiting	3	10%	
Education Level (*n* = 30)			
Associates Degree	4	13.3%	
Bachelor’s Degree	4	13.3%	
Grade 10–12	18	60%	
Grade 7–9	1	3.3%	
Other	3	10%	
Number of Children *	28		2.29; 1.18
Race/Ethnicity (*n* = 31)			
Black	10	32.3%	
Hispanic	5	16.1%	
White	12	38.7%	
Multiracial	3	9.7%	
Other	1	3.2%	
Age of Home (*n* = 30)			
Before 1950	8	26.7%	
Between 1950–1978	3	10%	
After 1978	1	3.3%	
Don’t know	18	60%	
Income Level ($) (*n* = 26)			
Less than 19,999	14	53.9%	
20,000–29,999	6	23.1%	
30,000–39,999	3	11.5%	
40,000–49,999	1	3.9%	
50,000–59,999	1	3.9%	
60,000–69,999	0	0.0%	
70,000–79,999	1	3.9%	
Residence Status (*n* = 29)			
Renting	23	79.3%	
Homeowner	3	10.3%	
Renting and Homeowner	3	10.3%	
Home painted with lead-based paint?			
Yes	5	17.9%	
No	8	28.6%	
Do not know	15	53.6%	
Observed any peeling, chipping, or cracking paint?			
Yes	16	57.1%	
No	12	42.9%	

* One participant reported caring for a grandchild and multiple other participants reported being pregnant. Grandchildren and unborn children were not included in the mean number of children or standard deviation (SD) calculations.

## Data Availability

The data presented in this study are available on request from the corresponding author. The data are not publicly available due to confidential agreement included in the consent form. Participants were assured that data would only be available to researchers on the team.

## Abbreviations

HUD	Department of Housing and Urban Development
WIC	Women, Infants and Children
LHC	Lead Hazard Control
ADHD	Attention Deficit Hyperactivity Disorder
